# Automated Tariff Classification of Organic Chemicals Using Molecular Fingerprints and Interpretable Structural Features

**DOI:** 10.3390/molecules31214036

**Published:** 2026-11-09

**Authors:** David Anderson

**Affiliations:** Joint Research Centre, European Commission, Retieseweg 111, Geel 2440, Belgium; david.anderson@ec.europa.eu

**Keywords:** combined nomenclature, cheminformatics, customs classification, machine learning, molecular fingerprints, tariff prediction

## Abstract

Tariff classification of chemical substances has traditionally relied on manual expert review, due to the need for in-depth chemistry knowledge and interpretation of complex rules. The volume of classification work is large and growing, and expert time is the limiting resource. This work investigates whether a machine learning classifier based on molecular structure can predict eight-digit tariff codes for organic chemicals, i.e., Chapter 29 of the Combined Nomenclature (CN). Labelled data for over 70,000 chemicals was downloaded from the European Customs Inventory of Chemical Substances (ECICS). Morgan fingerprints combined with other structural features were used to train three different machine learning algorithms. The best performance was achieved with the XGBoost algorithm: top-1 accuracy on a held-out 20% test set was 97.0% for the four-digit heading and 94.3% for the eight-digit CN code. For top-3 accuracy, the figures increased to 99.6% and 99.0%, respectively. For the eight-digit classifier, these figures do not include codes with fewer than 50 substances, for which a separate approach was used. The combined end-to-end performance was a top-1 accuracy of 91.2% and a top-3 accuracy of 97.9%. End-to-end performance on a temporal test set (not available at any time during model development) was similar: a top-1 accuracy of 90.3% and a top-3 accuracy of 97.3%.

## 1. Introduction

The Harmonized Commodity Description and Coding System, generally referred to as the Harmonised System (HS), is a multipurpose international product nomenclature developed by the World Customs Organization (WCO). It comprises more than 5000 commodity groups, each identified by a six-digit code, arranged in a legal and logical structure and supported by extensive rules aimed at ensuring uniform classification. The system is used by more than 200 countries and economies as a basis for their customs tariffs and for the collection of international trade statistics. Over 98% of the merchandise in international trade is classified in terms of the HS [1]. In the European Union (EU), the system is extended to eight digits as the Combined Nomenclature (CN) [2]. The CN subheadings stated in declarations for imported and exported goods determine which rate of customs duty applies and how the goods are treated for statistical purposes or for other EU policies [3]. In 2024, EU exports of chemicals and related products to non-EU countries were a record €560 billion, while the value of imports of chemicals was €322 billion [4].

Within Section VI of the CN (Products of the Chemical or Allied Industries), there are 11 chapters spanning over 80 pages. Chapter 29 (organic chemicals) is the largest of these, spanning 27 pages, and it also accounts for around 78% of entries in the ECICS (see Section 3.1). Classification of substances according to Chapter 29 is a very technical and time-consuming task requiring expert knowledge of organic chemistry and knowledge of the legal text and interpretative rules. It is necessary to identify a substance’s precise chemical structure, functional groups and sometimes its origin or method of manufacture, then apply a strict hierarchy of legal notes.

For example, Note 1 defines the scope of the whole chapter, so before classifying what something is, an expert first has to judge whether it legally qualifies as “separate chemically defined compounds”, given a specific list of allowed exceptions. Note 4 draws chemical distinctions, e.g., nitro/nitroso groups are not “nitrogen-functions” for heading 2929, even though structurally they are obviously nitrogen-containing groups. Note 5 lays out an entire sub-system for esters, salts, alcoholates, acid halides and coordination compounds, several of which are reassigned to whichever of two constituent headings occurs last in numerical order. At the subheading level, some codes single out one individually named compound (2912 42 00 is ethylvanillin), while others bundle several named substances into a single code (2917 39 20 groups a tetrabromophthalic ester, benzene-tricarboxylic acid, isophthaloyl dichloride, a naphthalene-tetracarboxylic acid and tetrachlorophthalic anhydride together).

Correctly navigating this combination of legal notes, chemical classification criteria and named exceptions is precisely why Chapter 29 classification has traditionally required trained customs chemists. By contrast, the aim of this study is to investigate the potential of cheminformatics and machine learning to automatically classify substances according to Chapter 29 based purely on their molecular structure. Chapter 29 was chosen because it accounts for around 78% of entries in the available dataset and because it is considered the chapter in which molecular structure has the greatest influence on classification.

In the scientific literature, there do not appear to be any published studies that address tariff classification of chemicals specifically. Most papers on automated classification concern general product classification or specific chapters that are not relevant for this work. Some related publications over the last ten years are presented below, with the aim of illustrating the evolution of the technical approaches and providing some indications of model performance.

Early approaches were rule-based, using text similarity approaches and classical machine learning (SVM, random forest) or graph/similarity methods over bag-of-words or TF-IDF text. Ding et al. [5] introduced Background Net—an incrementally learned, co-occurrence graph of terms proposed as an alternative to the vector space model for handling an “open and evolving vocabulary”. On Singapore customs data, it reached ~91% top-1 accuracy on Chapter 22 (beverages, spirits and vinegar) but only ~53% on Chapter 90 (optical instruments and apparatus), a shortfall traced to short, generic descriptions lacking disambiguating detail. Turhan et al. [6] described one of the first models to combine both text and visual features from product images, reaching a top-10 accuracy above 80% for four-digit HS Codes. Altaheri and Shaalan [7] benchmarked six classical algorithms on 218,000 cleaned Dubai Customs records; linear SVM performed best (76.3% full-code, 84.6% four-digit). Paramartha et al. [8] replicated this pipeline on Indonesian customs data and showed that language-specific preprocessing (custom dictionaries, spelling correction, translation) drove an ~11-point improvement (random forest: 79.6% F1 at four digits). Spichakova and Haav [9,10] reframed the task as auditing an already declared code rather than predicting one from scratch. On the 1.2-million-row U.S. Bill of Lading dataset, ~80% of declared codes received the top correctness score.

As larger labelled datasets became available, the field shifted to deep learning, where feature representations are learned instead of being hand-crafted. Li and Li [11] trained parallel text and image CNNs for Chinese cross-border e-commerce, fusing their outputs with a per-class adaptive weight; on a four-class footwear dataset, fusion (93.8%) marginally beat text alone (93.4%) and clearly beat images alone (76.8%). Binh et al. [12] addressed the HS hierarchy directly with a self-attention model producing four parallel outputs (chapter, heading, subheading, national extension) on 751,328 Vietnamese declarations (1167 labels). The key finding was architectural: a flat classifier collapsed at scale (micro-F1 0.044) while hierarchical decomposition degraded far more gracefully (0.64). He et al. [13] fused a Bidirectional Encoder Representations from Transformers (BERT)—a language model originally released by Google in 2018—with a BERT-embedding-plus-CNN hybrid, beating classical ML (SVM/RF/XGBoost) on two Chinese customs datasets (up to 99.4% weighted-F1 on the cleaner one). Zhou et al. [14] combined a Shallow Structured CNN with an Auxiliary Network identifying which fields discriminate confusable sub-categories. On Chinese customs data (up to 1 M records), it beat BERT-type approaches by ~3 F1 points with ~25× fewer parameters.

A number of approaches are based on multimodal and knowledge graph techniques. These combine product text with additional structure—images or a knowledge graph encoding relationships between codes and product attributes—on the premise that text alone is an incomplete signal. Amel et al. [15] fused product images (ResNet50/ViT/CLIP) with three text fields (SimCSE embeddings); adding images improved top-1 accuracy by 8.2 points over text alone, though the marginal value of images shrank once multiple text fields were already combined. Arya et al. [16] built a three-stage ensemble—hierarchical BERT, cosine similarity comparison and a knowledge graph extracted from WCO tariff manual text. Hierarchical BERT gained 16 points over a flat classifier and, crucially, 100% coverage vs. 88%. Qi et al. [17] generalised this with “KBGAT,” a graph attention network treating HS prediction as knowledge graph link completion, beating BERT by a wide margin (83.4% vs. 60.2%) in multi-category settings.

Finally, some approaches leverage pre-trained large language models instead of training a text model from scratch [18,19,20]. With a view to testing whether general-purpose large language models (LLMs), rather than purpose-built classifiers, can correctly apply the HS’s hierarchical rules, Yang et al. [21] introduced “HSCodeComp,” a benchmark of 632 real, deliberately noisy e-commerce listings (27 chapters). The best configuration tested reached only 46.8% exact match accuracy, against a 95.0% human expert ceiling.

These publications treat HS/CN classification as a general-purpose product classification problem. The specific domains studied—footwear, electronics, beverages and cargo manifests spanning arbitrary chapters—are singled out for size or value. One dataset [14] mentions “chemical raw materials” only in passing among many other chapter types. The closest related work to this study is a commercial product, ExpediChem by Scitegrity [22], which performs essentially the same task as this study, but—critically—it is a hand-crafted, rule-based structure matching system that does not use machine learning [23].

To the author’s best knowledge, this work is the first study to use molecular structure and machine learning to predict tariff codes of chemicals. The approach is to resolve substance names (IUPAC names, trade names, synonyms) to canonical SMILES, then derive structural features to train classical machine learning algorithms. Using the XGBoost algorithm, a top-1 accuracy of 94.3% and a top-3 accuracy of 99.0% were achieved for eight-digit CN codes (excluding classes with fewer than 50 samples). The approach described in this study is intended to serve as a decision support tool for customs classification of organic chemicals.

## 2. Results and Discussion

### 2.1. Accuracy on the Held-Out 20% Test Set

The dataset downloaded from ECICS in April 2026 yielded 71,601 substances—each with a SMILES notation, CN code and unique Customs Union and Statistics (CUS) number. Following a stratified 80:20 train/test split of the dataset, three algorithms were trained and tested. The overall accuracy of the four-digit and eight-digit classifiers is given in Table 1 and Table 2, respectively. Accuracy is defined as the fraction of all predictions that were correct, and the top-N accuracy is the fraction of instances where the true class was somewhere in the model’s top N predictions. The k-NN model was trained only on the Morgan fingerprints and MACCS keys and can be considered a cheminformatics baseline. The random forest and XGBoost models were trained on the full set of features. For both the four- and eight-digit classifiers, the best performance was obtained by the XGBoost algorithm.

### 2.2. Classification Report

More insight into the performance can be obtained from the classification report. Table 3 shows the classification report for the four-digit XGBoost classifier. Precision is defined as the fraction of predicted labels X that were actually X. Recall is the fraction of true labels Y that were predicted as Y. F1-score is the harmonic mean of precision and recall: a single number that penalises either low precision or low recall. Support is the number of true examples of each class in the test set.

Performance across most Chapter 29 headings was excellent, with the majority of precision, recall and F1 scores above 0.95. The overall accuracy was 0.97, and the macro average—where every class is treated equally regardless of support—was 0.95. The clear exceptions are headings 2936 to 2942, with f1-scores between 0.67 and 0.88.

The reason for the lower performance on these headings is that they are defined not purely by chemical structure, but partly by biological origin or function: 2936 (vitamins), 2937 (hormones), 2939 (alkaloids) and 2941 (antibiotics). Two molecules can be near-identical in skeleton (same scaffold encoded in very similar Morgan fingerprints) yet sit in different headings because one is classified by its physiological role rather than its structure. A structure-only model has no way to see “used as a hormone”. Heading 2940 (sugars, chemically pure, other than sucrose etc.) is partly defined by exclusion: the heading text explicitly carves out “other than products of headings 2937, 2938 or 2939.” So, a molecule’s correct heading can depend on whether it is already claimed by a neighbouring heading, rather than a positive structural feature of the molecule itself. Finally, heading 2942 is the catch-all “other organic compounds” bucket, with a support of just N = 5. Low support alone makes precision/recall unstable (one or two errors swing the score wildly).

Although the top-1 accuracy of headings 2936 to 2941 was notably lower than the macro average of 0.95, the correct heading was almost always recovered in the second or third prediction (see Table 4). The strong top-2 and top-3 performance therefore suggests that the model has learned the relevant chemical relationships but sometimes assigns the highest probability to a closely related structural class. For example, some vitamins, hormones and alkaloids contain nitrogen heterocycles and may therefore resemble compounds of heading 2933, and sugars share structural features with oxygen heterocycles (2932) and glycosides (2938).

The classification report for the eight-digit classifier is reported in Appendix A.

### 2.3. Exploring Top Features

To verify that the classifier’s predictions reflect genuine structural reasoning rather than incidental correlations in the training data, the features contributing the most to the model’s decision for each class were determined using Tree SHAP [24,25], computed natively via XGBoost’s pred_contribs mechanism.

**Figure 1:**
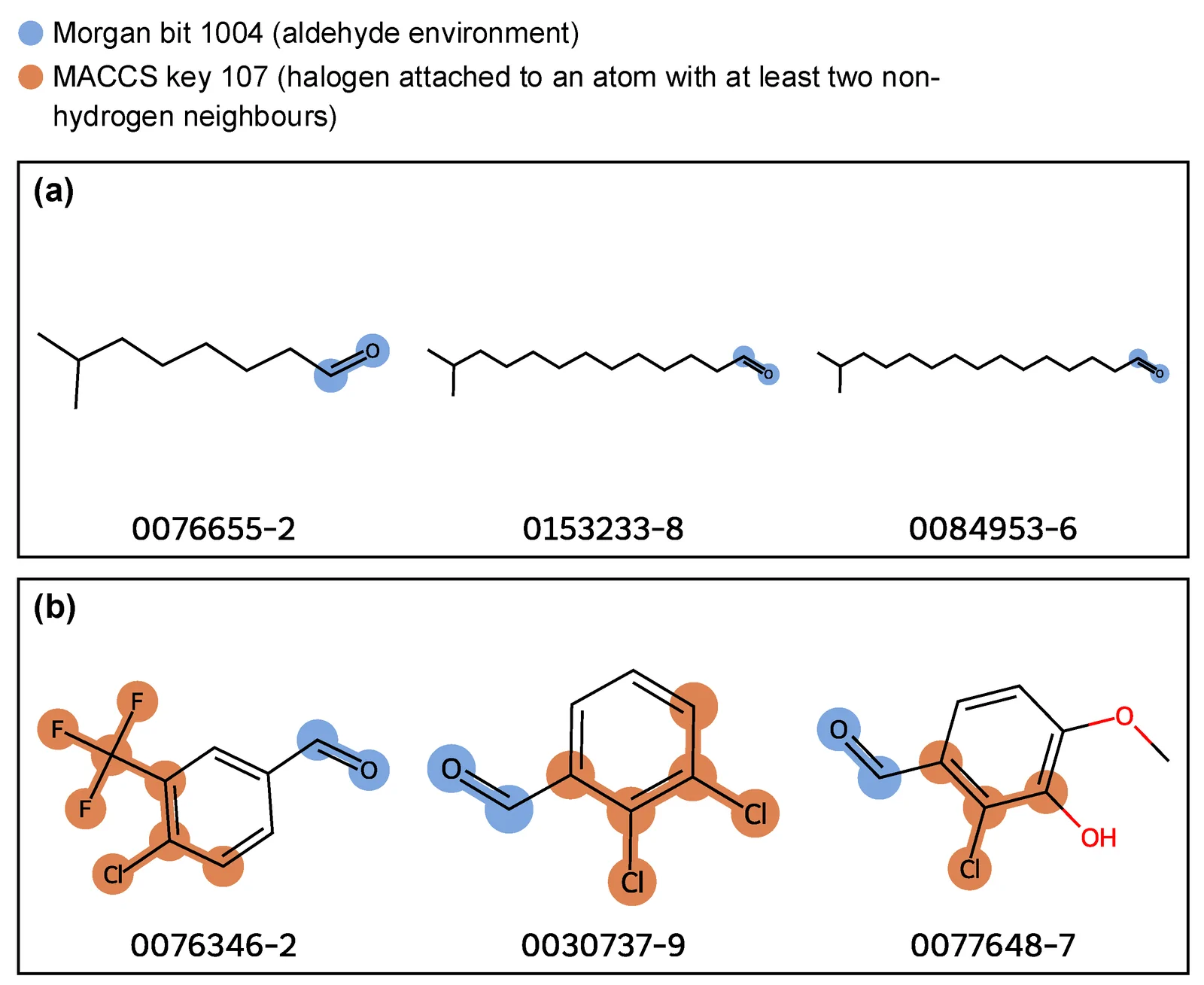
Example molecules and their CUS numbers to illustrate the top structural features used by the XGBoost model to predict two classes: (**a**) 2912 (aldehydes): the presence of Morgan fingerprint bit 1004 (aldehyde-carbon environment); (**b**) 2913 (halogenated/sulphonated/nitrated/nitrosated derivatives): presence of Morgan bit 1004 and of MACCS keys 107/134 (halogens).

In the first example, we note the top structural features used to predict classes 2912 and 2913, i.e., aldehydes, vs. their halogenated/sulphonated/nitrated/nitrosated derivatives. The dominant feature in both is a Morgan fingerprint bit that captures the aldehyde-carbon environment. The presence or absence of MACCS keys 107 and 134 (halogens) helps the model to discriminate between the two classes (see Figure 1).

**Figure 2:**
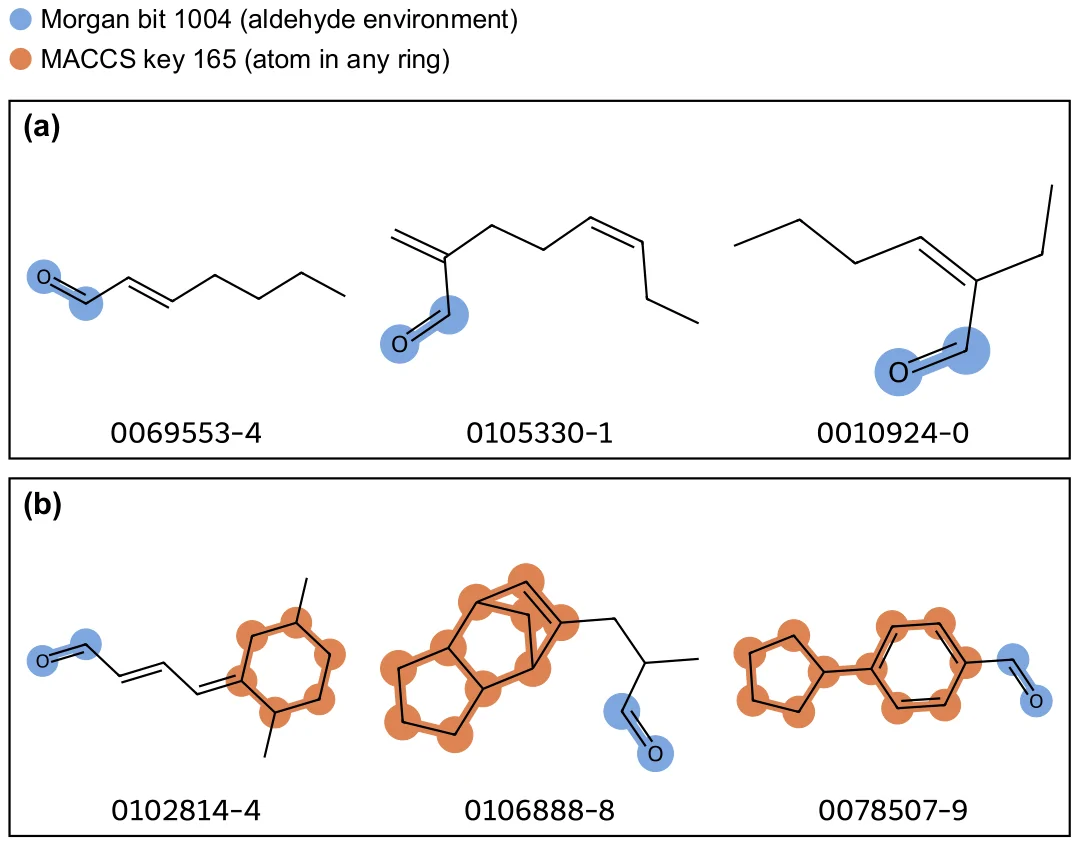
Example molecules and their CUS numbers to illustrate the top structural features used by the XGBoost model to predict two classes: (**a**) 2912 19 00 (acyclic aldehydes): the presence of Morgan fingerprint bit 1004 (aldehyde-carbon environment); (**b**) 2912 29 00 (cyclic aldehydes): presence of Morgan bit 1004 and of MACCS key 165 (ring structure).

The second example is for eight-digit classes under the same four-digit parent (2912), namely acyclic aldehydes (2912 19 00) and cyclic aldehydes (2912 29 00). Similar to the previous example, Morgan fingerprint bit 1004 (aldehyde-carbon environment) is present in both, but MACCS key 165 (ring structure) is present only in 2912 29 00 (see Figure 2).

For reference, the top-10 features for the four classes in Figure 1 and Figure 2 are listed in Appendix B. Knowing which features are driving a model’s predictions for each individual class is a very powerful capability that greatly facilitates explainable AI. It helps move from a “black-box” model of the classifier to something that users can comprehend and trust. The two examples above illustrate that the model is using precisely the structural features a customs chemist would expect. The XGBoost model, however, is doing more than just checking for the presence or absence of structural patterns; it is considering the combined signal of many weighted features and constructing a series of internal decision trees in a manner that optimises the model’s performance across a large training set.

### 2.4. Performance vs. Similarity

**Figure 3:**
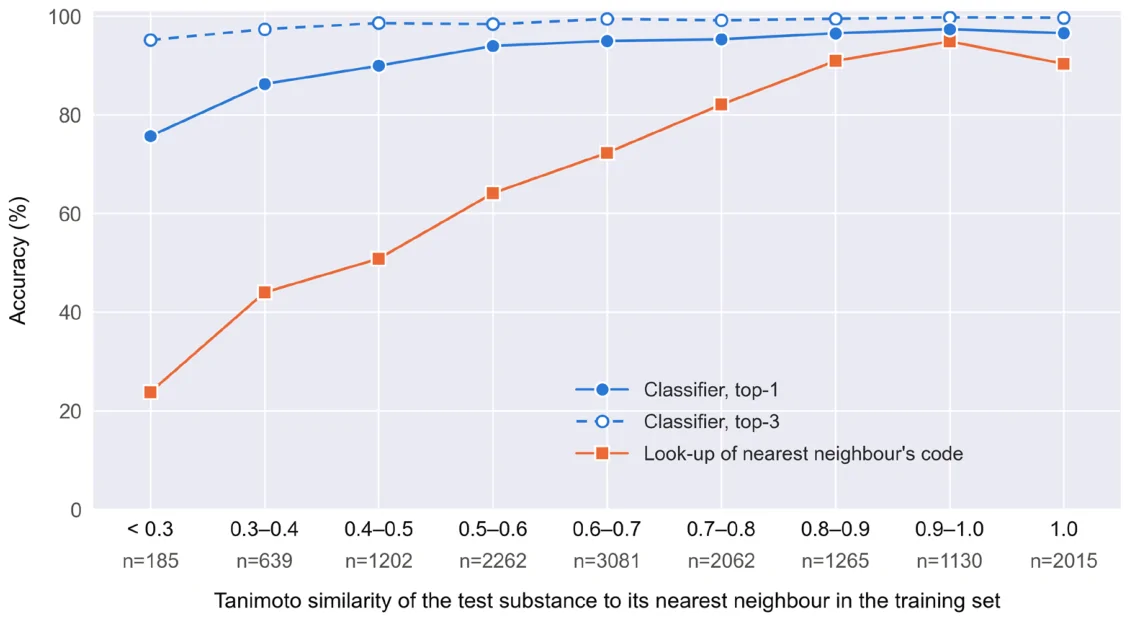
Top-1 and top-3 accuracy (rare classes excluded) of the eight-digit classifier and of a look-up of the nearest neighbour’s code in each similarity range on the 20% held-out test set.

Considering the large number of substances in the dataset, it is possible that some molecules in the test set are very structurally similar to molecules in the training set. In order to have reassurance that the model is not simply memorising structures, the relationship between model accuracy and similarity to the training set was analysed. The similarity of each test substance to the training data was quantified as its Tanimoto similarity to the most similar substance in the training set using the same Morgan fingerprints as the model. The test substances of the eight-digit classifier (rare classes excluded) were then grouped by bins of nearest neighbour similarity, and the accuracy was calculated for each group (Figure 3). As a baseline, the accuracy was calculated also for a simple look-up that assigns each test substance the CN code of its nearest neighbour. If classification were simply a matter of looking up known structures, the two approaches would perform similarly.

As can be seen in Figure 3, the classifier substantially outperforms the simple look-up across the whole test set. Over all 13,841 test substances, it assigns the correct eight-digit code to 94.3%, compared with 74.8% for the look-up, an improvement of almost 20 percentage points. The advantage is greatest precisely where there is no substance with a high similarity. For substances without a close analogue in the training data (similarity below 0.4), the look-up identifies the correct code with a top-1 accuracy of around 24–44%, whereas the classifier achieves around 76–86%. The classifier therefore does not simply recognise structures it has already seen: it has learnt the relationships between structural features and classification and applies them successfully even to substances unlike those in the training data.

Conversely, the reported accuracy is not inflated by near-identical structures shared between the training and test sets. Excluding all test substances that have a close analogue in the training set (similarity of 0.9 or above, 22.7% of the test set) changes the top-1 accuracy only from 94.3% to 93.6%.

### 2.5. End-to-End Evaluation, Including Rare Classes

As described in the Data and Methods section, the classifier was trained only on the 150 eight-digit codes with at least 50 substances in the dataset. A parallel approach based on simple structural similarity was used to flag potentially rare classes (Section 3.5). This section reports the performance of the complete workflow, combining the two approaches. Whenever a rare class is flagged, the best-matching rare class replaces the third prediction of the classifier, so that exactly three codes are always proposed, and top-3 accuracy can be calculated end to end. On the full test set of 14,321 substances (including 480 substances belonging to rare classes), the combined top-3 accuracy was 97.9%. The breakdown is given in Table 5.

### 2.6. Temporal Test: Substances Added to ECICS After Model Development

The model was also evaluated on a temporal test set of 10,259 Chapter 29 substances that were added to ECICS between the original data download (April 2026) and October 2026. The classifier developed on the 80% training data was frozen and applied exactly as reported in the preceding sections, with no retraining and no changes to the settings. For this temporal test set, the top-1 accuracy for eight-digit CN codes was 93.2% (compared with 94.3% on the held-out test set), whilst top-3 accuracy was 98.9% (compared with 99.0% on the held-out test set).

**Figure 4:**
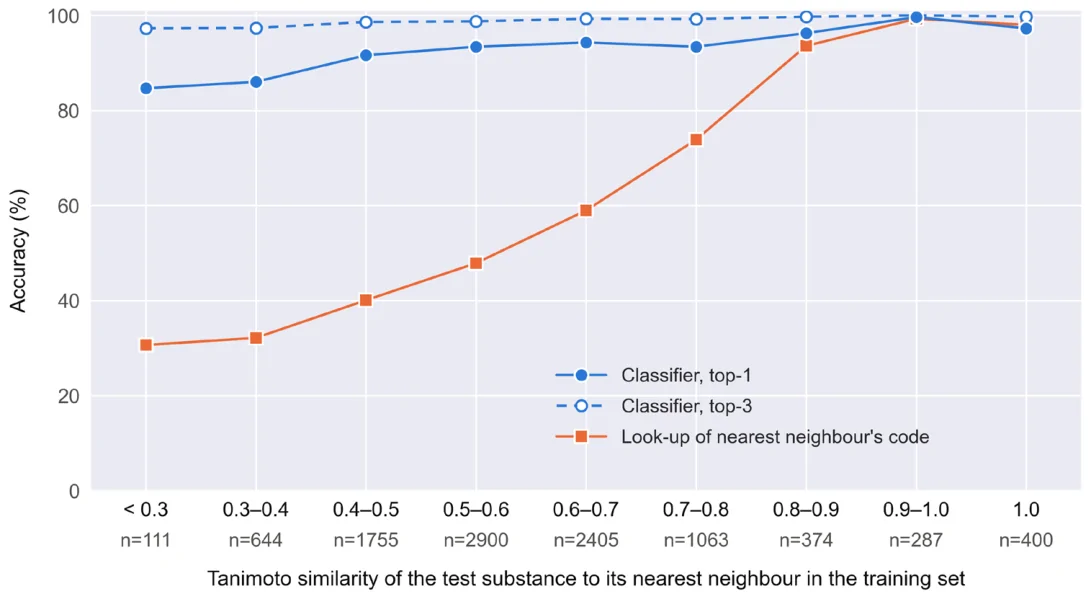
Top-1 and top-3 accuracy (rare classes excluded) of the eight-digit classifier and of a look-up of the nearest neighbour’s code in each similarity range on a temporal test set (not available at any stage of model development).

Figure 4 shows the performance of the classifier on the temporal test set against similarity to the training data, and also the baseline performance of the simple look-up of the nearest neighbour code. At a given similarity, the classifier has a similar accuracy to that of the held-out 20% test set (see Figure 3). For the temporal test set, the gap between the classifier performance and the simple look-up of the nearest neighbour’s code (e.g., 93% against 48% for similarity range 0.5–0.6) was even more pronounced than for the held-out test set. This is strong evidence that the classifier has learnt general relationships rather than relying on close neighbours.

The performance of the complete workflow that combines the classifier and the rare-class flagging was also evaluated for the temporal test set. As in Section 2.5, whenever a rare class was flagged, the best-matching rare class replaced the third prediction of the classifier, so that exactly three codes are always proposed, and top-3 accuracy could be calculated end to end. On the complete temporal test set of 10,259 substances, the top-1 accuracy for eight-digit CN codes was 90.3%, and the top-3 accuracy was 97.3%. The breakdown is given in Table 6.

### 2.7. Machine Learning vs. Human Evaluation

Beyond accuracy metrics, it is worth comparing how a machine-learning-based classifier compares with traditional human-based classification more broadly. Table 7 compares both approaches across seven criteria. These reflections further support the positioning of the classifier as a decision support tool that augments, rather than replaces, human expertise—handling the high-volume, structurally well-defined majority of cases quickly and consistently, while flagging the edge cases or rare classes (see Section 3.5) for expert review.

In addition to supporting the classification of new substances, an AI-based classifier can support the maintenance and quality control of existing inventories. Any large classification database, compiled by many experts over many years and under a nomenclature that changes periodically, will contain a degree of inconsistency. Because the AI model can re-assess an entire inventory in seconds, it can flag entries for expert review: for example, structurally identical or closely related substances with different codes, entries where the model disagrees with high confidence, or entries likely to be affected by amendments to the CN. These can be ranked so that expert time is directed where it is most useful. Related work has explored the use of classifiers for auditing of previously declared tariff codes [9,10].

## 3. Data and Methods

### 3.1. European Customs Inventory of Chemical Substances (ECICS)

**Figure 5:**
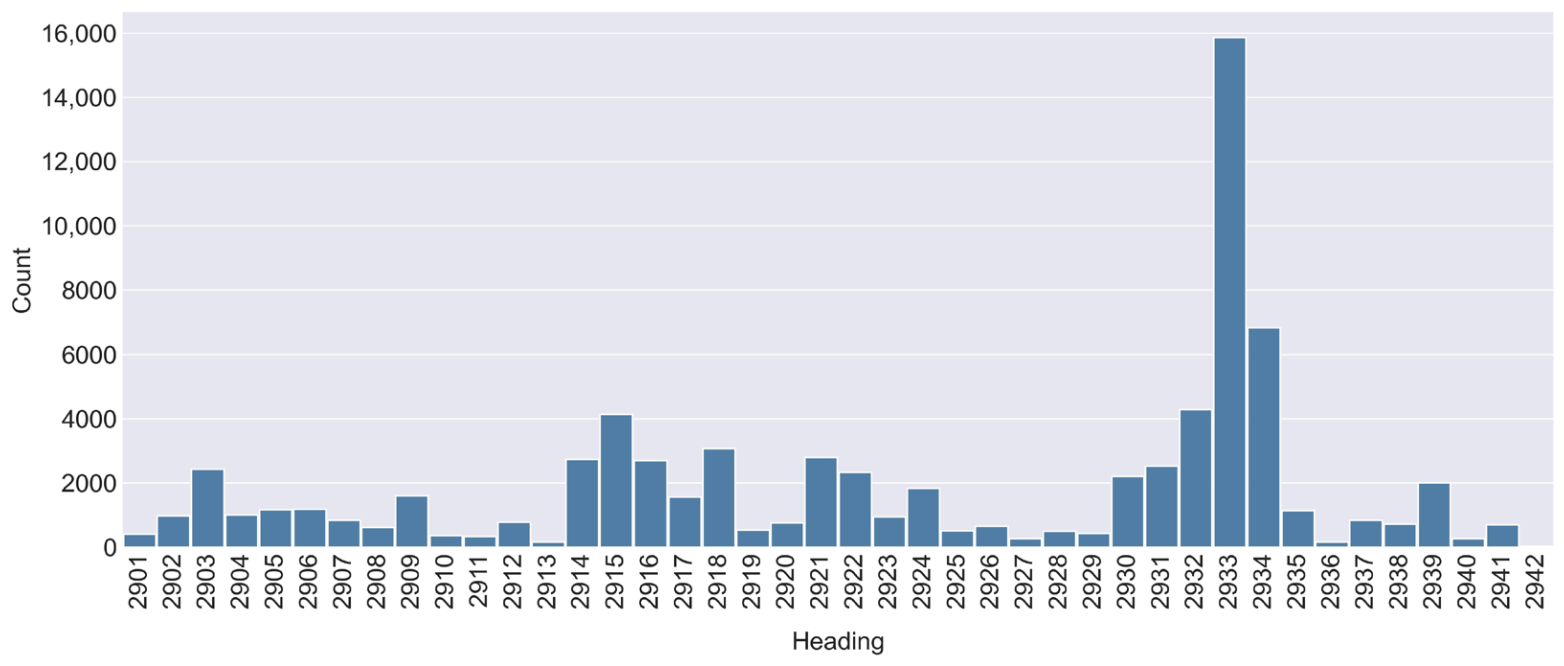
Distribution of 75,081 substances in the ECICS dataset across the 42 headings in Chapter 29 (organic chemicals) as downloaded in April 2026.

To perform supervised learning, a dataset of chemical substances labelled by subject matter experts with CN codes was downloaded from the ECICS as an Excel file in April 2026 [26]. This file contained 96,016 substances—each indexed with a unique CUS number. Around 78% of entries belong to Chapter 29 (organic chemicals). Within Chapter 29, there are 42 classes with a four-digit HS code (referred to as headings). The distribution of ECICS data across these 42 headings is shown in Figure 5.

Within Chapter 29, there are 523 unique eight-digit codes—referred to as CN codes. A total of 10 codes had no substance with a resolvable structure, leaving 513 codes. The most common CN code contained 5357 substances, whilst at the other extreme, there were 183 CN codes that contained just 1 substance. The large number of these “rare classes” is an important consideration for the subsequent step of machine learning. It is impossible to learn a class based on just one substance. Even classes with 10 or 20 substances are difficult to learn. For this reason, the author decided to apply a threshold; classes with fewer than 50 substances (3.6% of the dataset) were set aside. With an 80:20 split, a class of 50 substances has about 40 training and 10 test examples. Below that, there are too few examples for the model to learn the class and too few test substances to measure its performance reliably (with five test substances, a single error changes the score by 20%). This threshold keeps 150 of the 513 codes and 96.4% of the substances. The remaining 363 codes contain only 3.6% of the substances, and these are handled by a separate approach to identify potential “rare classes” (see Section 3.5). An overview of the data flow at the various steps is given in Appendix C.

**Figure 6:**
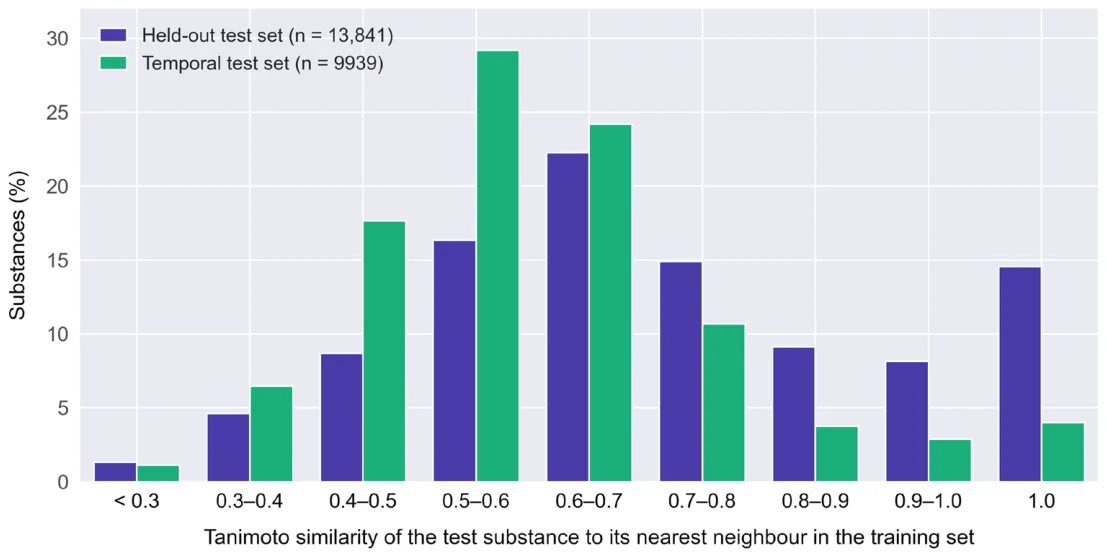
Comparison of the held-out 20% test set (April 2026) and the temporal test set (October 2026) in terms of similarity to the training data.

Data from ECICS was downloaded again in October 2026, and the dataset contained an additional 10,918 Chapter 29 substances that were added to ECICS between April and October 2026. These substances were therefore not available at any stage of model development. Their CN codes were assigned via the normal procedure (human classification followed by review by EU Member States’ experts). Of the 10,259 substances for which OPSIN returned a SMILES, 320 belonged to rare classes and 9939 to non-rare classes (mainly in headings 2922 to 2931). The composition of the temporal test set and the 20% held-out test set are compared in Figure 6 in terms of Tanimoto similarity to the nearest neighbour in the training set. We can see that the temporal test set is less similar to the training data than the held-out 20% test set: 25.3% of substances have a similarity below 0.5 (against 14.6% in the held-out set), whilst 6.9% of substances have a similarity of 0.9 or above (against 22.7% in the held-out set). This temporal test set therefore provides a valuable opportunity to test the classifier on unseen chemicals.

### 3.2. SMILES Generation

In order to generate molecular fingerprints of chemical substances using the RDKit (version 2026.3.1) library in Python (version 3.13), it was first necessary to obtain a SMILES notation for each substance. SMILES stands for Simplified Molecular Input Line Entry System, and it is a specification in the form of a line notation for describing the structure of chemical species using short ASCII strings. One of the most common and reproducible ways of getting SMILES from IUPAC names is OPSIN—which stands for Open Parser for Systematic IUPAC Nomenclature [27]. For this work involving tens of thousands of chemicals, it was preferable to use a local implementation of OPSIN rather than slower web services. The py2opsin library [28] is a Python wrapper around OPSIN. As a local process, it can process large batches of chemicals quickly, i.e., milliseconds per molecule, compared with around 5 s per molecule individually. In this work, version 2.8.0 of OPSIN jar was used (included in version 1.1.0 of py2opsin). In order to extract as many SMILES notations as possible, a Python script was written to pre-process all the chemical names, batch convert them to SMILES, and then select one entry per CUS number according to a predefined priority order.

It was not possible, however, to generate SMILES notations for every substance in ECICS. The reason for this is that OPSIN expects chemical names (ideally IUPAC names) as input and is quite strict about the correct notation. Furthermore, some entries in ECICS have only common names or synonyms, for example, “soapbark extract” or “heavy fuel oil”. In these cases, the terms do not describe a specific molecule. Using OPSIN, SMILES were generated for 90% of the substances in Chapter 29.

Additional SMILES were obtained using PubChem—the open chemistry database at the National Institutes of Health [29]. A Python script was created to query the database programmatically using a REST-style gateway. Via PubChem, SMILES were obtained for an additional 4130 unique CUS numbers—thereby increasing the SMILES generation rate for Chapter 29 from 90% to 95%. The final length of the dataset after RDKit was 71,601 substances in Chapter 29—each with a SMILES notation, CN code and unique CUS number.

### 3.3. Feature Engineering

To generate features based on molecular structure, version 2026.03.1 of the RDKit library was used [30]. The SMILES generated previously were each used to create a Mol object—which is the internal representation of a molecule in RDKit. For each substance, a total of 2240 features were created, as summarised in Table 8.

Morgan fingerprints were created using RDKit’s in-built GetMorganGenerator function. Morgan fingerprints represent a molecule as a fixed-length vector based on the local environment of each atom. Starting from each atom, the algorithm builds identifiers for increasingly larger neighbourhoods by incorporating nearby atoms and bonds up to a specified radius. These identifiers are then hashed to positions in the vector, producing a compact representation of molecular structure that can be used for similarity searching or machine learning. In addition to molecular fingerprints, additional features were created.

In addition to Morgan fingerprints, features were created using Molecular ACCess System (MACCS) keys. These are a set of 166 predefined structural fragments for substructure and similarity searching [31]. Each molecule is represented as a 166-bit binary vector, where each bit indicates the presence (1) or absence (0) of a specific structural feature—for example, whether the molecule contains an aromatic ring, a carbon–halogen bond, more than one nitrogen atom or a carbonyl group [32]. Unlike Morgan fingerprints, which encode circular atom neighbourhoods and can represent an effectively unbounded structural vocabulary, MACCS keys are a fixed, human-interpretable set of chemically meaningful substructures.

### 3.4. Machine Learning

For this study, three different machine learning algorithms were trained and tested: k-nearest neighbours (k-NN), random forest (RF) and extreme gradient boosting (XGBoost). k-NN is a simple, non-parametric classification method that can find the most structurally similar substances in the training set and take a majority vote among them. For molecules, similarity is conventionally measured using the Tanimoto coefficient, which for binary fingerprint bit vectors is equivalent to the Jaccard index. For this reason, only the Morgan fingerprint (2048 bits) and MACCS key (167 bits) blocks of the feature matrix were used. Tanimoto-similarity k-NN is one of the oldest and most widely used baselines in cheminformatics. It is a relatively simple reference point against which to judge whether more expressive models like random forest or XGBoost are actually adding value.

Random forest is an ensemble of decision trees built independently and in parallel, each on a bootstrap sample of the data and a random subset of features at each split. Predictions are averaged (or majority-voted). Trees are typically grown deep/unpruned—individually high-variance, low-bias—and averaging across many decorrelated trees is what cancels out that variance.

XGBoost is, by contrast, an ensemble of decision trees built sequentially, where each new tree is trained to correct the errors (technically, fit the negative gradient of the loss) left by the trees before it. The final prediction is a weighted sum of all trees’ outputs, not a vote. It adds explicit regularisation (L1/L2 penalties on leaf weights, tree complexity terms) and a learning rate that shrinks each tree’s contribution, both aimed at controlling overfitting from that sequential correction process. The XGBoost classifier holds back 10% of the training data as a validation set. It is used to decide when to stop adding trees (early stopping) and is never used to evaluate the model. A summary of the main settings of the three algorithms used in this study is given in Table 9.

After training and testing the classifiers, it was possible to extract the relative importance of the features. Table 10 reports the relative importance of each feature family for the XGBoost classifier using gain. Gain is XGBoost’s native importance metric and is defined as the average improvement in the boosting loss contributed by splits on that feature summed across all trees.

We can see from Table 10 that Morgan fingerprints and MACCS keys are the most important features. Compared with the four-digit classifier, the eight-digit classifier relies more on Morgan fingerprints. This is to be expected, as the four-digit classifier separates broad functional classes (e.g., heterocyclic compounds with nitrogen heteroatoms, carboxylic acids and amine-function compounds), which can be adequately described using relatively coarse features such as MACCS. For the eight-digit classifier, two molecules can have identical MACCS features and still belong to different classes, because what separates them is finer: exact chain length, substitution pattern, ring fusion details etc. Morgan fingerprints encode circular substructure neighbourhoods in much higher resolution (2048 bits vs. 166 MACCS keys), so they have more discriminative power and their share increases (from 77% to 93%). While the remaining features might not seem significant in terms of overall accuracy, they can be very important to resolve specific classes. The data and Python code used to create the features and perform the training and evaluation are provided as Supplementary Material for this article.

### 3.5. Strategy for Rare Classes

As described in Section 3.1, classes with fewer than 50 substances (3.6% of the dataset) were not used to train the classifier. For these rare classes, a separate strategy was implemented using a similarity search. For each query substance, the Tanimoto similarity (Morgan fingerprints and MACCS keys) to every substance in the training set was computed. A rare class was flagged if its most similar training substance was at least as similar to the query as the most similar training substance in any of the classifier’s top-3 predicted classes, less a margin. If one or more rare classes were flagged, the most similar one replaced the classifier’s third prediction, so that the user is always presented with exactly three codes.

**Figure 7:**
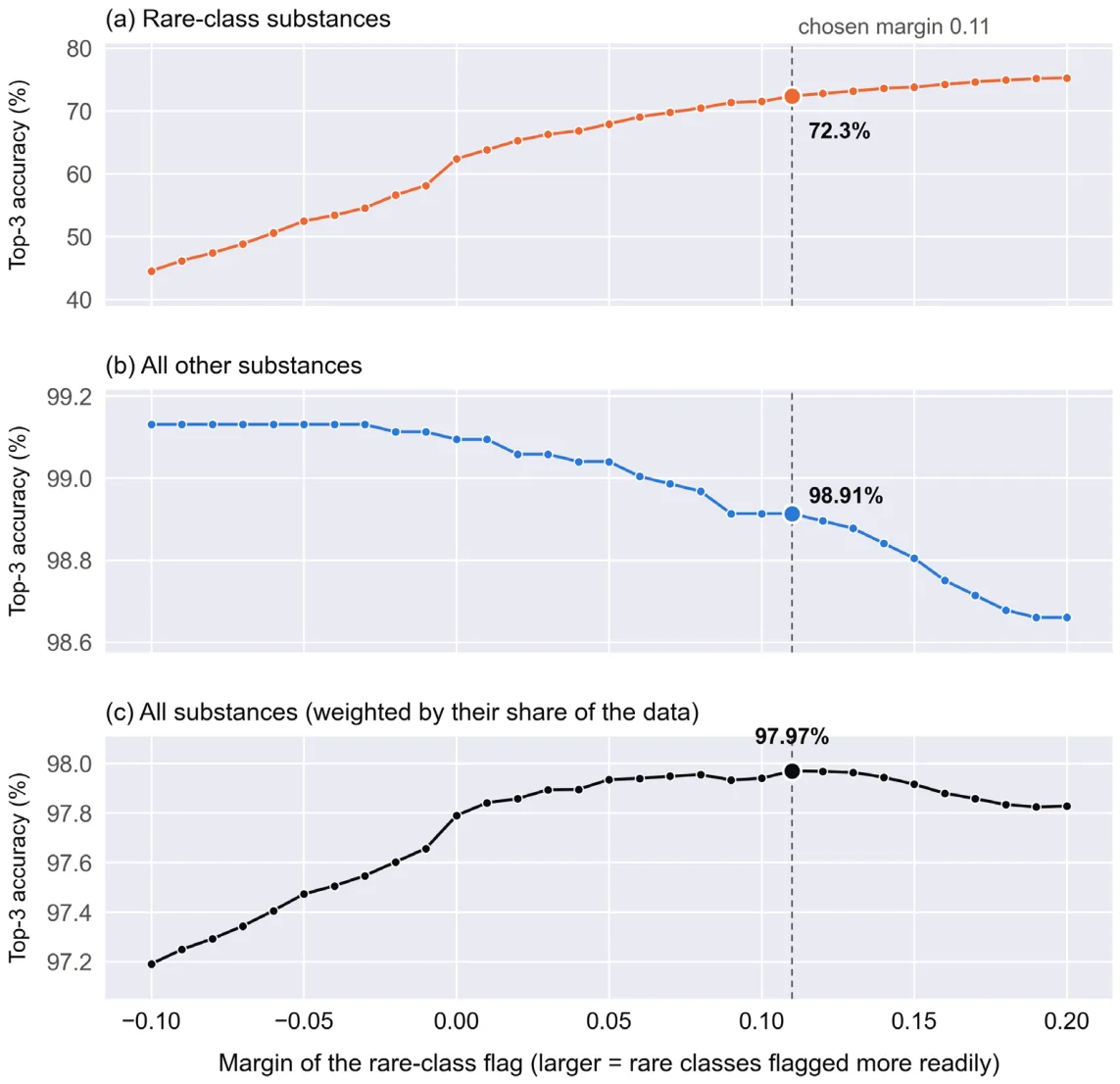
Effect of the rare-class margin on top-3 accuracy: (**a**) rare-class substances; (**b**) other substances; (**c**) all substances, weighted by their share of the dataset. The dashed line marks the chosen margin (0.11).

The margin was tuned on training data only: the 2063 rare-class substances in the training set, each compared with all other training substances, and the 5522 substances of the validation split. A larger margin flags rare classes more readily, so more rare-class substances have their code in the shortlist but correct third predictions are replaced more often (Figure 7). The chosen margin, 0.11, maximises top-3 accuracy over all substances, with rare-class and other substances weighted by their share of the dataset. The value of 0.11 is not critical: any margin from 0.05 to 0.14 gives a weighted top-3 accuracy within 0.05 percentage points of the maximum.

## 4. Conclusions

A machine learning classifier based only on molecular structure was developed to predict tariff codes of organic chemicals under Chapter 29 of the Combined Nomenclature (CN). Using the XGBoost algorithm, a top-1 accuracy of 94.3% and a top-3 accuracy of 99.0% were achieved for eight-digit CN codes, excluding classes with fewer than 50 samples. Performance is consistently high across headings 2901 to 2935. For headings 2936 to 2941, the top-1 accuracy was lower than the macro average of 0.95, but the correct heading was almost always recovered in the second or third prediction. This is because the classification is based on more than structure, i.e., application or origin. Using Tree SHAP analysis, it is possible to elucidate the individual features that contribute the most to the prediction of specific classes. For classes containing too few entries for the classifier to learn, a parallel approach was developed. The end-to-end performance of the classifier combined with the rare-class approach was a top-1 accuracy of 91.2% and a top-3 accuracy of 97.9% on the 20% held-out test set. Performance on a temporal test set of substances not available at any time during model development was similar, with an end-to-end top-1 accuracy of 90.3% and top-3 accuracy of 97.3%. The intended use is that the classifier is deployed on a batch of chemicals pending classification, and the top three predictions (including possible rare classes) are presented to a human expert for the final decision. The approach can also be used to check the consistency of existing inventories of classifications that have been built up over many years.

## Figures and Tables

**Table 1:** Comparison of accuracy of three ML models in predicting the four-digit heading. The test set comprised 14,321 substances.

Metric	k-NN	Random Forest	XGBoost
top-1 accuracy	0.884	0.959	0.970
top-2 accuracy	0.959	0.990	0.993
top-3 accuracy	0.969	0.994	0.996

**Table 2:** Comparison of accuracy of three ML models in predicting the eight-digit CN code. The test set comprised 13,841 substances (classes with fewer than 50 samples were excluded).

Metric	k-NN	Random Forest	XGBoost
top-1 accuracy	0.807	0.919	0.943
top-2 accuracy	0.910	0.970	0.981
top-3 accuracy	0.935	0.983	0.990
macro f1	0.772	0.898	0.929

**Table 3:** Classification report of XGBoost classifier for four-digit headings.

Heading	Label	Precision	Recall	F1-Score	Support
2901	Acyclic hydrocarbons	1.00	1.00	1.00	78
2902	Cyclic hydrocarbons	1.00	1.00	1.00	192
2903	Halogenated derivatives of hydrocarbons	1.00	1.00	1.00	482
2904	Sulphonated, nitrated or nitrosated derivatives of hydrocarbons, whether or not halogenated	0.99	0.99	0.99	198
2905	Acyclic alcohols and their halogenated, sulphonated, nitrated or nitrosated derivatives	0.99	1.00	0.99	228
2906	Cyclic alcohols and their halogenated, sulphonated, nitrated or nitrosated derivatives	0.99	0.99	0.99	227
2907	Phenols; phenol-alcohols	0.99	0.99	0.99	165
2908	Halogenated, sulphonated, nitrated or nitrosated derivatives of phenols or phenol-alcohols	0.99	0.98	0.98	122
2909	Ethers, ether-alcohols, ether-phenols, ether-alcohol-phenols, alcohol peroxides, ether peroxides, acetal and hemiacetal peroxides, ketone peroxides (whether or not chemically defined) and their halogenated, sulphonated, nitrated or nitrosated derivatives	1.00	0.98	0.99	314
2910	Epoxides, epoxyalcohols, epoxyphenols and epoxyethers with a three-membered ring and their halogenated, sulphonated, nitrated or nitrosated derivatives	1.00	1.00	1.00	67
2911	Acetals and hemiacetals, whether or not with other oxygen function, and their halogenated, sulphonated, nitrated or nitrosated derivatives	0.96	1.00	0.98	66
2912	Aldehydes, whether or not with other oxygen function; cyclic polymers of aldehydes; paraformaldehyde	0.99	0.99	0.99	153
2913	Halogenated, sulphonated, nitrated or nitrosated derivatives of products of heading 2912	0.94	1.00	0.97	32
2914	Ketones and quinones, whether or not with other oxygen function, and their halogenated, sulphonated, nitrated or nitrosated derivatives	0.97	0.97	0.97	516
2915	Saturated acyclic monocarboxylic acids and their anhydrides, halides, peroxides and peroxyacids; their halogenated, sulphonated, nitrated or nitrosated derivatives	0.98	0.97	0.98	799
2916	Unsaturated acyclic monocarboxylic acids and cyclic monocarboxylic acids, their anhydrides, halides, peroxides and peroxyacids; their halogenated, sulphonated, nitrated or nitrosated derivatives	0.98	0.98	0.98	530
2917	Polycarboxylic acids, their anhydrides, halides, peroxides and peroxyacids; their halogenated, sulphonated, nitrated or nitrosated derivatives	0.94	0.97	0.95	306
2918	Carboxylic acids with additional oxygen function and their anhydrides, halides, peroxides and peroxyacids; their halogenated, sulphonated, nitrated or nitrosated derivatives	0.98	0.98	0.98	595
2919	Phosphoric esters and their salts, including lactophosphates; their halogenated, sulphonated, nitrated or nitrosated derivatives	0.95	1.00	0.98	100
2920	Esters of other inorganic acids of non-metals (excluding esters of hydrogen halides) and their salts; their halogenated, sulphonated, nitrated or nitrosated derivatives	0.93	0.96	0.95	145
2921	Amine-function compounds	0.99	0.98	0.99	545
2922	Oxygen-function amino-compounds	0.98	0.97	0.97	458
2923	Quaternary ammonium salts and hydroxides; lecithins and other phosphoaminolipids, whether or not chemically defined	0.99	0.98	0.99	182
2924	Carboxyamide-function compounds; amide-function compounds of carbonic acid	0.97	0.99	0.98	360
2925	Carboxyimide-function compounds (including saccharin and its salts) and imine-function compounds	0.87	0.97	0.91	99
2926	Nitrile-function compounds	0.96	0.98	0.97	128
2927	Diazo-, azo- or azoxy-compounds	0.86	0.94	0.90	47
2928	Organic derivatives of hydrazine or of hydroxylamine	0.92	0.92	0.92	95
2929	Compounds with other nitrogen function	0.94	0.95	0.95	83
2930	Organo-sulphur compounds	0.97	1.00	0.99	354
2931	Other organo-inorganic compounds	0.98	0.95	0.97	476
2932	Heterocyclic compounds with oxygen hetero-atom(s) only	0.97	0.98	0.98	834
2933	Heterocyclic compounds with nitrogen hetero-atom(s) only	0.99	0.97	0.98	3049
2934	Nucleic acids and their salts, whether or not chemically defined; other heterocyclic compounds	0.97	0.98	0.98	1277
2935	Sulphonamides	0.98	1.00	0.99	225
2936	Provitamins and vitamins, natural or reproduced by synthesis (including natural concentrates), derivatives thereof used primarily as vitamins and intermixtures of the foregoing, whether or not in any solvent	0.71	0.82	0.76	27
2937	Hormones, prostaglandins, thromboxanes and leukotrienes, natural or reproduced by synthesis; derivatives and structural analogues thereof, including chain modified polypeptides, used primarily as hormones	0.77	0.82	0.80	146
2938	Glycosides, natural or reproduced by synthesis, and their salts, ethers, esters and other derivatives	0.82	0.94	0.88	64
2939	Alkaloids, natural or reproduced by synthesis, and their salts, ethers, esters and other derivatives	0.84	0.86	0.85	374
2940	Sugars, chemically pure, other than sucrose, lactose, maltose, glucose and fructose; sugar ethers, sugar acetals and sugar esters, and their salts, other than products of heading 2937, 2938 or 2939	0.90	0.78	0.83	45
2941	Antibiotics	0.89	0.80	0.84	133
2942	Other organic compounds	0.75	0.60	0.67	5
accuracy				0.97	14,321
macro average		0.94	0.95	0.95	14,321
weighted average		0.97	0.97	0.97	14,321

**Table 4:** Top-1 to top-3 accuracy (on 14,321 test samples) for headings 2936 to 2941 for the four-digit classifier using XGBoost.

Heading	*n*	Top-1 Accuracy	Top-2 Accuracy	Top-3 Accuracy
2936	27	0.81	0.96	0.96
2937	146	0.82	0.97	1.00
2938	64	0.94	0.95	0.95
2939	374	0.86	0.98	0.99
2940	45	0.78	0.93	0.98
2941	133	0.80	0.92	0.95

**Table 5:** End-to-end evaluation of the classifier combined with the rare-class flag on the test set.

Data	Support	Top-1 Accuracy	Top-2 Accuracy	Top-3 Accuracy
non-rare classes	13,841	0.943	0.981	0.988
rare classes ^1^	480	0.000	0.000	0.729
all test data	14,321	0.912	0.948	0.979

^1^ Because a rare class can only enter the shortlist in third position, the top-1 and top-2 accuracy for rare-class substances is always zero.

**Table 6:** End-to-end evaluation of the classifier and the rare-class flag on the temporal test set.

Data	Support	Top-1 Accuracy	Top-2 Accuracy	Top-3 Accuracy
non-rare classes	9939	0.932	0.979	0.985
rare classes ^1^	320	0.000	0.000	0.600
all test data	10,259	0.903	0.948	0.973

^1^ Because a rare class can only enter the shortlist in third position, the top-1 and top-2 accuracy for rare-class substances is always zero.

**Table 7:** Comparison of machine-learning- and human-based customs classification.

Criterion	Machine Learning	Human Judgement
Speed	Near-instantaneous per substance; once trained, the classifier can evaluate 14,000 chemicals in ten seconds	Minutes to hours per case, especially for structurally complex or borderline substances
Scalability/cost at volume	Marginal cost per prediction is near-zero once trained	Cost scales roughly linearly with volume, requiring proportionally more trained staff
Consistency	Deterministic; once model is trained, substances always receive identical predictions	Subject to variability, fatigue and drift in judgement over time or between evaluators, although consensus voting partially addresses this
Adaptability to rule changes or reclassification	The classifier can be easily and quickly re-trained in the event of changes to the tariff classification rules and/or reclassification of historical entries	Updating hand-crafted rules or retraining human evaluators would require significantly more effort
Handling non-structural criteria	Weaker where classification depends on biological origin, intended use or legal exclusion notes rather than molecular structure	Human experts can directly incorporate contextual, functional or legal knowledge that a structure-only model has no access to
Novel/out-of-distribution substances	Reliability depends on similarity to training examples; genuinely novel chemistries may be poorly represented	Can reason from first-principles chemical and legal knowledge even for substances never previously encountered
Transparency and accountability	Historically a “black box,” though interpretability methods such as the Tree SHAP analysis presented here (Section 2.3) help address this	Rulings are attributable to a named, accountable expert who can explain and defend a decision, e.g., in a customs dispute

**Table 8:** Overview of 2240 features used to train the random forest and XGBoost classifiers.

Feature Family	Feature Count	Description
Morgan fingerprints	2048	Circular substructure fingerprint, radius 2, hashed to a 2048-bit vector
MACCS keys ^1^	167	RDKit’s standard 166-key structural dictionary (predefined substructure patterns)
Physicochemical	8	MolWt, LogP, TPSA, H-bond donors, H-bond acceptors, rotatable bonds, ring count, fraction sp3 carbon
Elemental	10	Atom counts for C, N, O, S, P, F, Cl, Br, I, plus total halogen count
Heterocycle	6	Heterocycle count, aromatic/saturated/aliphatic ring counts, spiro atoms, bridgehead atoms
Max chain length	1	Longest carbon-only path in the molecule

^1^ Comprises 166 data columns plus one zero-indexed empty column.

**Table 9:** Summary of key settings and parameters used for the k-NN, random forest and XGBoost classifiers.

Characteristic	k-NN	Random Forest	XGBoost
Features	Morgan FP + MACCS keys (2215 binary columns)	Full feature matrix (2240 columns)	Full feature matrix (2240 columns)
Key parameters	n_neighbours = 5	n_estimators = 300; max_depth unconstrained	n_estimators = 300 (early stopping); max_depth = 6; learning_rate = 0.1
Class imbalance handling	none	class_weight = “balanced”	Sample weights computed (“balanced”)
Regularisation/stopping	-	-	Early stopping (20 rounds) on a 10% held-out validation split

**Table 10:** Ranking of feature families after training four-digit and eight-digit XGBoost classifiers. Values are shown to two significant figures.

Feature Family	Feature Ranking	
	Four-Digit	Eight-Digit
Morgan FP	77%	93%
MACCS keys	22%	6.1%
Elemental	0.20%	0.31%
Heterocycle	0.52%	0.15%
Physicochemical	0.43%	0.11%
Max chain length	0.025%	0.024%

**Figure A1:**
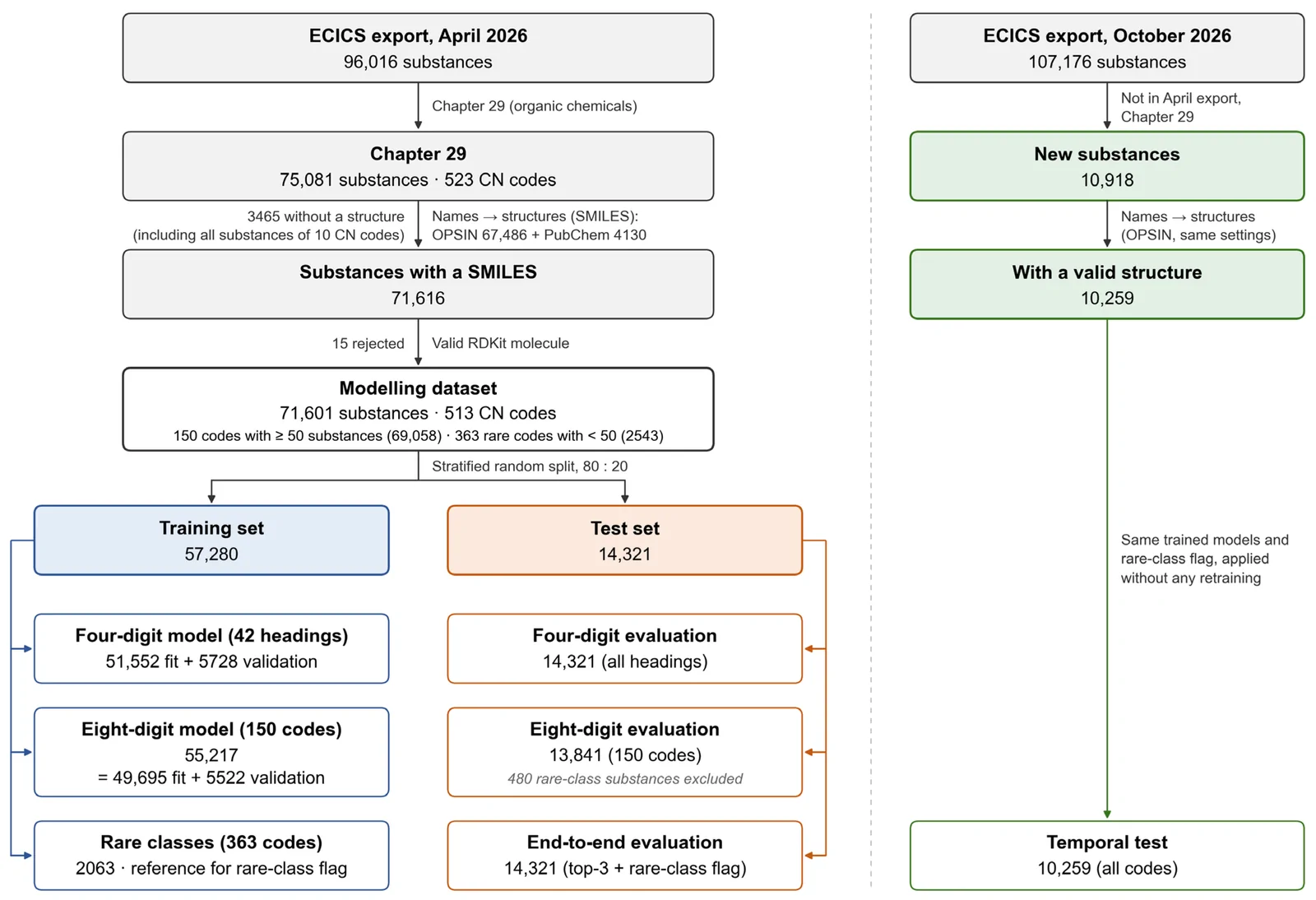
Overview of data flow. On the left is the preparation of the modelling dataset from April 2026, showing the 80:20 train:test split, the rare classes and the end-to-end workflow. On the right is the temporal test set from October 2026, which was evaluated with the same model.

## Data Availability

Data used in this study was downloaded here: http://ec.europa.eu/taxation_customs/dds2/ecics/chemicalsubstance_consultation.jsp?Lang=en (accessed on 23 September 2026).

## Supplementary Materials

The following supporting information can be downloaded at: https://www.mdpi.com/article/10.3390/molecules31214036/s1.

## Abbreviations

The following abbreviations are used in this manuscript:

AI	artificial intelligence
ASCII	American standard code for information interchange
BERT	bidirectional encoder representations from transformers
CN	Combined Nomenclature
CNN	convolutional neural network
CUS	Customs Union and Statistics
ECICS	European Customs Inventory of Chemical Substances
EU	European Union
GenAI	generative artificial intelligence
HS	Harmonized System
IUPAC	International Union of Pure and Applied Chemistry
k-NN	k-nearest neighbours
LLM	large language model
MACCS	molecular access system
ML	machine learning
OPSIN	open parser for systematic IUPAC nomenclature
RF	random forest
SMILES	simplified molecular input line entry system
SVM	support vector machine
TF-IDF	term frequency—inverse document frequency
WCO	World Customs Organization
XGBoost	extreme gradient boosting

## Appendix A. Classification Table for Eight-Digit Classifier on Held-Out Test Set

**Table A1:** Classification report of the eight-digit XGBoost classifier on the held-out test set (13,841 substances; the 150 CN codes with at least 50 substances; rare classes excluded).

CN Code	Precision	Recall	F1	Support
2901 10 00	1.00	1.00	1.00	28
2901 29 00	1.00	1.00	1.00	50
2902 19 00	0.97	1.00	0.98	63
2902 90 00	0.99	0.99	0.99	127
2903 19 00	1.00	1.00	1.00	22
2903 29 00	1.00	1.00	1.00	16
2903 59 00	1.00	1.00	1.00	22
2903 69 19	1.00	1.00	1.00	32
2903 77 90	1.00	0.85	0.92	13
2903 79 30	0.98	1.00	0.99	102
2903 89 70	1.00	1.00	1.00	10
2903 99 80	0.99	1.00	1.00	204
2904 10 00	1.00	1.00	1.00	83
2904 20 00	0.96	0.96	0.96	28
2904 99 00	0.93	0.99	0.96	85
2905 19 00	0.94	1.00	0.97	64
2905 29 90	0.98	0.98	0.98	54
2905 39 95	0.97	1.00	0.99	35
2905 49 00	1.00	1.00	1.00	11
2905 59 98	0.98	0.95	0.96	43
2906 13 10	0.81	1.00	0.90	13
2906 19 00	0.99	0.96	0.97	136
2906 29 00	0.99	1.00	0.99	75
2907 13 00	0.90	0.90	0.90	10
2907 19 90	0.99	0.97	0.98	81
2907 29 00	0.98	1.00	0.99	65
2908 19 00	1.00	1.00	1.00	56
2908 99 00	1.00	0.95	0.97	62
2909 19 90	0.98	0.98	0.98	55
2909 30 39	0.91	1.00	0.95	20
2909 30 90	0.96	0.98	0.97	84
2909 49 80	1.00	0.97	0.98	94
2909 50 00	0.94	0.94	0.94	31
2910 90 00	0.98	1.00	0.99	66
2911 00 00	0.96	0.98	0.97	66
2912 19 00	1.00	1.00	1.00	55
2912 29 00	1.00	1.00	1.00	50
2912 49 00	0.96	0.96	0.96	47
2913 00 00	0.91	0.94	0.92	32
2914 19 90	1.00	0.98	0.99	67
2914 29 00	0.94	0.94	0.94	101
2914 39 00	0.97	0.99	0.98	71
2914 40 90	0.81	0.88	0.84	40
2914 50 00	0.97	0.95	0.96	62
2914 69 80	0.88	1.00	0.94	22
2914 79 00	0.96	0.95	0.95	147
2915 13 00	0.93	0.96	0.94	27
2915 29 00	1.00	0.90	0.94	19
2915 39 00	0.98	0.96	0.97	192
2915 40 00	1.00	1.00	1.00	9
2915 50 00	0.91	0.95	0.93	21
2915 60 19	0.95	1.00	0.98	63
2915 60 90	0.89	0.98	0.93	48
2915 70 40	0.86	0.75	0.80	24
2915 70 50	0.64	0.90	0.75	20
2915 90 30	0.71	0.81	0.76	21
2915 90 90	0.96	0.92	0.94	346
2916 12 00	0.89	0.98	0.93	47
2916 14 00	0.94	0.98	0.96	52
2916 15 00	0.84	1.00	0.91	32
2916 19 95	0.94	0.94	0.94	107
2916 20 00	0.97	0.97	0.97	60
2916 31 00	0.97	1.00	0.98	29
2916 39 90	0.97	0.96	0.97	166
2917 11 00	0.89	0.94	0.91	17
2917 12 00	0.63	0.80	0.71	15
2917 13 90	0.78	0.82	0.80	17
2917 19 80	0.97	0.94	0.95	125
2917 20 00	0.97	1.00	0.99	35
2917 34 00	0.85	0.92	0.89	25
2917 39 85	0.93	0.95	0.94	66
2918 11 00	1.00	0.95	0.97	20
2918 13 00	0.96	0.96	0.96	23
2918 15 00	0.95	1.00	0.97	18
2918 19 98	0.90	0.89	0.90	112
2918 23 00	1.00	1.00	1.00	13
2918 29 00	0.91	1.00	0.95	74
2918 30 00	0.96	0.93	0.95	106
2918 99 90	0.95	0.92	0.94	209
2919 90 00	0.96	1.00	0.98	100
2920 29 00	0.93	0.93	0.93	29
2920 90 10	0.93	0.95	0.94	59
2920 90 70	0.98	1.00	0.99	48
2921 19 99	0.99	0.95	0.97	95
2921 29 00	1.00	0.96	0.98	49
2921 30 99	0.93	0.96	0.94	26
2921 42 00	0.98	0.92	0.95	51
2921 43 00	0.97	1.00	0.98	28
2921 45 00	0.96	1.00	0.98	25
2921 49 00	0.97	0.97	0.97	134
2921 51 19	0.77	1.00	0.87	10
2921 51 90	0.72	0.68	0.70	19
2921 59 90	0.92	0.87	0.89	63
2922 19 00	0.93	0.94	0.93	125
2922 29 00	0.95	0.96	0.95	93
2922 39 00	0.83	0.93	0.88	27
2922 49 85	0.99	0.95	0.97	102
2922 50 00	0.93	0.89	0.91	85
2923 90 00	0.99	0.98	0.98	170
2924 19 00	0.94	0.99	0.97	99
2924 21 00	0.95	1.00	0.98	21
2924 29 70	0.97	0.98	0.97	239
2925 19 95	0.68	0.89	0.77	36
2925 29 00	0.91	0.97	0.94	61
2926 90 70	0.96	0.98	0.97	128
2927 00 00	0.90	0.92	0.91	47
2928 00 90	0.91	0.94	0.92	94
2929 10 00	0.95	0.95	0.95	21
2929 90 90	0.88	0.94	0.91	62
2930 20 00	0.92	0.92	0.92	12
2930 90 16	0.75	0.75	0.75	12
2930 90 95	0.97	0.96	0.97	327
2931 49 80	0.98	0.97	0.98	199
2931 59 90	0.98	0.97	0.98	107
2931 90 00	0.97	0.90	0.93	154
2932 19 00	0.97	0.97	0.97	110
2932 20 90	0.95	0.98	0.97	286
2932 99 00	0.96	0.96	0.96	431
2933 19 90	0.97	0.97	0.97	177
2933 21 00	0.91	1.00	0.95	20
2933 29 90	0.93	0.96	0.95	192
2933 34 00	0.72	0.95	0.82	19
2933 39 99	0.96	0.92	0.94	692
2933 49 10	0.74	0.88	0.80	16
2933 49 90	0.84	0.91	0.88	115
2933 54 00	0.93	0.96	0.94	26
2933 59 95	0.89	0.94	0.91	432
2933 69 80	0.95	0.98	0.96	195
2933 79 00	0.80	0.91	0.85	196
2933 99 80	0.97	0.89	0.93	926
2934 10 00	0.95	0.94	0.94	128
2934 20 80	0.91	0.98	0.94	92
2934 30 90	1.00	0.99	0.99	70
2934 99 90	0.96	0.94	0.95	979
2935 90 90	0.98	0.99	0.99	220
2937 19 00	0.79	0.83	0.81	36
2937 22 00	0.79	0.92	0.85	24
2937 23 00	0.63	0.61	0.62	31
2937 29 00	0.56	0.58	0.57	38
2937 50 00	0.91	0.77	0.83	13
2938 90 90	0.87	0.90	0.88	58
2939 11 00	0.63	0.86	0.73	14
2939 19 00	0.76	0.76	0.76	29
2939 59 00	0.79	0.96	0.87	28
2939 69 00	0.87	1.00	0.93	13
2939 79 90	0.87	0.82	0.85	205
2939 80 00	0.85	0.66	0.74	53
2940 00 00	0.88	0.78	0.82	45
2941 10 00	0.95	1.00	0.97	19
2941 90 00	0.89	0.74	0.81	98
Accuracy			0.943	13,841
Macro average	0.920	0.940	0.929	13,841
Weighted average	0.945	0.943	0.943	13,841

## Appendix B. Top 10 Features of XGBoost Classifier for Selected Classes

**Table A2:** Top 10 features for class 2912 determined using Tree SHAP.

Rank	Feature	Signed Mean SHAP	Class Prevalence
1	Morgan bit 1004	4.965	99.30%
2	MACCS key 134: [F, Cl, Br, I]	0.573	0.00%
3	TPSA	0.402	100.00%
4	Morgan bit 694	0.313	99.30%
5	MACCS key 161: [#7]	0.245	0.00%
6	Morgan bit 1414	0.215	0.00%
7	MACCS key 123: [#8]~[#6]~[#8]	0.145	0.70%
8	Morgan bit 1917	0.125	1.30%
9	LogP	0.118	94.10%
10	MolWt	0.115	100.00%

**Table A3:** Top 10 features for class 2913 determined using Tree SHAP.

Rank	Feature	Signed Mean SHAP	Class Prevalence
1	Morgan bit 1004	5.630	100.00%
2	MACCS key 107	0.643	75.00%
3	MACCS key 134: [F, Cl, Br, I]	0.594	78.10%
4	Morgan bit 694	0.362	100.00%
5	Morgan bit 1063	0.266	87.50%
6	MACCS key 63: [#7]=[#8]	0.198	18.80%
7	Morgan bit 1057	0.165	21.90%
8	MACCS key 123: [#8]~[#6]~[#8]	0.159	0.00%
9	MACCS key 129: [$(*~[CH2]~*~*~[CH2]~*),$([R]1@[CH2]@[R]@[R]@[CH2;R]1),$(*~[CH2]~[R]1@[R]@[CH2;R]1)]	0.130	0.00%
10	MACCS key 161: [#7]	0.103	18.80%

**Table A4:** Top 10 features for class 2912 19 00 determined using Tree SHAP.

Rank	Feature	Signed Mean SHAP	Class Prevalence
1	Morgan bit 1004	5.686	100.00%
2	TPSA	0.799	100.00%
3	MACCS key 165: [R]	0.728	0.00%
4	Morgan bit 401	0.530	56.40%
5	HBA	0.485	100.00%
6	MACCS key 157: [#6]-[#8]	0.292	0.00%
7	max_carbon_chain	0.261	100.00%
8	MACCS key 153	0.233	0.00%
9	MACCS key 152: [#8]~[#6](~[#6])~[#6]	0.197	0.00%
10	RotatableBonds	0.175	100.00%

**Table A5:** Top 10 features for class 2912 29 00 determined using Tree SHAP.

Rank	Feature	Signed Mean SHAP	Class Prevalence
1	Morgan bit 1004	5.780	100.00%
2	TPSA	1.945	100.00%
3	MACCS key 165: [R]	1.010	100.00%
4	Morgan bit 694	0.859	100.00%
5	MACCS key 134: [F, Cl, Br, I]	0.532	0.00%
6	MolWt	0.186	100.00%
7	Morgan bit 926	0.139	56.00%
8	MACCS key 163	0.135	92.00%
9	MACCS key 161: [#7]	0.123	0.00%
10	Morgan bit 807	0.120	22.00%
